# Observations on Scarlatina Anginosa, as It Appeared at St. Clairsville, Ohio, in 1833

**Published:** 1842-11

**Authors:** Thomas Carroll

**Affiliations:** Now of Cincinnati


### Art. III.—*Observations on Scarlatina Anginosa, as it appeared at St. Clairsville, Ohio, in* 1833. By Thomas Carroll, M. D., now of Cincinnati.

Before I bring to the reader's consideration the disease which is the principal object of this essay, I will make a few observations on the anginose affections, with scarlatina, that preceded the year 1833.

My own observation will not enable me to say much about those diseases anterior to the year 1829; and *nothing* farther back than 1824. From that year until 1829 scarlatina was not observed by me, but angina maligna occasionally prevailed, and often proved fatal.

During the winter of 1828—9 scarlatina simplex first made its appearance at St. Clairsville, and in a few localities in its neighborhood. During that winter I did not lose a single case, though the efflorescence in most cases was general, and somewhat intense in its character. Indeed, my success was such as to inspire me with great confidence in the treatment of that too often unmanageable disease; but alas! time soon brought down my arrogance, and taught me, that, like epidemic cholera, this disease often sets at defiance the best directed efforts of our art.

From this period until 1833, scarlatina almost constantly prevailed, either at St. Clairsville or in various surrounding localities, as well as at different places in the belt of country lying between the Ohio and the Muskingum rivers, where it occasioned great mortality, though at St. Clairsville but very few fatal cases occurred. Indeed that town and its vicinity has at no time suffered with the same severity as many other places around it. Of those who died in 1833 many were adults, among whom were persons of such note in society as to attract attention to the prevailing mortality, and perhaps make it seem greater than it really was.

This epidemic of 1833 is that to which I desire to direct the reader's attention, as its character was different from any type of scarlatina I have ever seen. It commenced in February, declined in July, but did not terminate until August. Its character changed with the state of the season, being more intense in hot than in cool weather. This remark will at least hold good with regard to those cases which originated in cool weather, which instantly got worse, if any considerable rise of the thermometer suddenly took place, especially if they had been sick from one to four or five days. So well did I understand this matter, that on one occasion, when a sudden change of temperature from cold to warm occurred, I predicted its sinister effect on my patients; and within a week I lost five adults in town. This fact, in connexion with others, warned me to avoid heated and close rooms in the treatment of this disease; indeed, I am not certain but heat is as injurious in the this disease as in small-pox. When scarlatina commences during hot weather, the cases often do well; or if it commence in warm, and terminate in cold weather, I think it is not aggravated by the change.

The first appearance of the disease in February was characterised by inflamed fauces, elevated and reddened papillæ of the tongue, with swelling of the mucous membrane generally, slight salivation, headache, rigors, fever and delirium. The fever, in most cases, was accompanied with an unusually strong and frequent pulse, and great heat of the surface, but almost altogether without eruption of the skin. This condition lasted from four to seven days; when, if the patient did well, the symptoms abated, and he was convalescent by the tenth day. When the result was unfavorable, a singular, and to me, new set of symptoms supervened with continued delirium, and other signs of inflammation of the brain.

As the season advanced, these symptoms became united with other and more serious ones, that frequently bade defiance to all the efforts made to stop their fatal tendency; and were mostly brought on by imprudence of the patients after they had pretty well recovered from the severity of the disease, by active treatment at the outset followed up by abstemiousness and quiet. When by these means the patient was so far restored as to go about the house and resume his usual habits, some irregularity in eating and drinking, or the exposure of himself to cold and wet, or even washing in cold water, has brought on the following symptoms: Fever, with a revival of the inflammation of the fauces, frequently travelling up the posterior nares, and involving the whole pituitary membrane, and the sub-mucous cellular substance; and at length attacking the skin above. The first appearance of inflammation of the last, was mostly observed on the bridge of the nose; the centre of it being at the termination of the ossa nasi, and forming a belt across the nose, about three fourths of an inch wide, with an apex resting on each cheek, though sometimes there was but one. This inflamed belt either crossed one or both cheeks; and when it only travelled across one side of the face it invaded but one ear; but if the contrary, both were involved in the difficulty. The swelling spread from this belt, and mostly the whole face and scalp became involved in the tumefaction; though, where the inflammation spread on one side of the nose and only crossed one cheek, but one side of the face swelled, and no part of the scalp inflamed. The color of the skin was red, more or less approaching a venous hue, and during a few of the first hours had a polished aspect: but vesication uniformly resulted in a short time, and spread over the face and ears, but did not invade the hairy scalp to much extent; which union of the vesication and swelling completed every characteristic of erysipelas, and was accordingly treated as such. Abscesses also formed about the head, and I know no instance of death when they arose.

This condition in those who survived it, lasted about ten days before the swelling materially moderated; when it gradually yielded, and the patient recovered his usual health; and, so far as I saw, no return of erysipelas took place. But nearly half of those who were thus diseased, never recovered; inflammation of the brain having eventually carried them off, and that mostly within the first week or ten days from the commencement of the erysipelas.

In most cases the lips and tongue swelled until articulation was either difficult or impossible, and the tumefaction extended over the face and scalp until the eyes were so buried beneath that vision was impossible. Abscesses frequently formed either in the upper eyelids or in the scalp, each of which contained about a tea-spoonful of pus when fully matured. It is singular that no case of death took place, as I have just said, when these abscesses formed about the head; which was not the case when they supervened on the extremities. Other and more appalling symptoms occurred in a number of cases, the result of which was the death of every one in whom they were developed. These consisted in swellings of the extremities that mostly terminated either in suppuration or mortification. They were confined to the cellular substance both exterior to, and beneath, the fascia surrounding the muscles; and in one or two instances, in the larger joints. In the cases which terminated in suppuration, healthy pus was not formed; but a thin darkish matter approaching to green, was the result. Amidst this matter there was, in nearly every case, a quantity of fibrin closely adhering together, and forming a substance more or less extensive according to the size of the abscess. Their common length was from three to six inches; their width one and a half or two, and their thickness from three to five lines. These substances adhered so closely together, that I generally succeeded in drawing them out of the orifices made for the exit of the matter. To give a clearer idea of the nature and effect of these abscesses, I subjoin the two following cases.

A gentleman, aged about 65, was taken in the usual way, and did not at first seem very ill. He would take no medicine, nor submit to depletion by bleeding, and indulged in eating whatever he wished. He continued to have fever after the time it usually subsided, and at the end of three weeks he suddenly complained of pains in the calves of the legs, which on examination were found to be swollen, particularly on the inner sides. Soon after, both his fore-arms inflamed and one of his elbows, and all went on to suppuration; abscesses forming on the inner side of each leg and arm, and one round the elbow joint. When the matter was discharged four fibrinous substances were drawn from four of them. After this, they all healed, and a sixth formed low down on the right side. This abscess was very large and extremely painful. It connected itself slowly with the walls of the abdomen, which rendered it easy to reach the matter with an instrument; but I long hesitated before I opened it, as I thought that death would soon follow the evacuation of the pus. The earnest solicitations of the patient, however, overcame my hesitation. I opened it, and my patient within seventy-two hours paid the debt of nature, after having suffered a most painful disease during three months. About ten days from the commencement of this patient's illness, his wife died of mortification of two of her extremities by the same disease.

A lady aged 35, had at the commencement the usual symptoms, and in the course of a week seemed to be rapidly recovering. She returned to her ordinary occupations, which exposed her too much. The febrile symptoms recurred, and the inner surface of each fore-arm inflamed and suppurated. I opened the abscesses, and drew out of each strips of fibrin. These abscesses eventually healed, and the patient again seemed to be doing well; but, of a sudden, pain and swelling of the side of the head, with fever and delirium set in; and before suppuration took place death ensued, in about five weeks from the first attack.

Five cases terminated in mortification. In two of the patients the arm was its seat; and in one of these it also attacked the leg. Three others had one leg each mortified. The first of those affected in the arm seemed to have had the mortification brought on by bleeding, as pain at the bend of the arm, with inflammation of the orifice, and swelling of the limb, took place within 24 hours after the operation. The patient lived only three days after the commencement of sphacelation, which within that time involved the greater part of the arm. In a few days afterwards, however, I was called to prescribe for the sister of this patient, a lady advanced to the 65th year of her age, though ten years younger than her brother. When I examined her, my impression was so strong that she must be bled, that I proposed taking blood; but my patient refused, on the ground that she thought her brother had fallen a victim to bleeding. My reasons for wishing to bleed were, that she had an uncommonly strong pulse, pain in the head, hot and dry skin, and soreness of the throat; but the repugnance of my patient, and the melancholy termination of the last case caused me to yield; and I made the best prescription I could under the circumstances. On the following day, to my great astonishment, I found the arm, out of which I had intended to draw blood, considerably swollen and very painful, and the leg of the same side in the same condition. The color of the limbs was slightly venous; indeed, venous congestion was very obvious. At my next visit, 24 hours after, I found both limbs affected with incipient mortification. Another day brought about a fatal termination.

In the three remaining cases, the arms escaped, and but one extremity was affected in each. Great pain was experienced in the inflammatory stage. This state of suffering lasted but a short time, probably not much over 36 hours, in any of the cases; when other symptoms, less painful but equally distressing, supervened. One of these cases was that of an intemperate colored man, about 35 years of age. Another that of a lady advanced to the sixtieth year of her age, whose constitution had been broken up by long continued disease. The third and last had sound health up to the time of her fatal attack; she was a poor and laborious woman. They all died.

The inflammation in the above cases was no doubt of the erysipelatous character; and its malignity arose from its close connection with a masked form of the scarlatina.

Not less than five pregnant females became affected with the epidemic of 1833, and one case of a similar kind occurred in 1834, of which all but one proved fatal about the fifth or sixth day.

The first case I saw in consultation. The lady had been ill three or four days, and had been taken with a violent rigor, which was succeeded by intense fever, accompanied by pain of the head, back, throat and chest; and after the continuance of this for some hours, another rigor followed, which was again succeeded by fever. When I first saw this patient I determined in my own mind that the death of the fœtus had taken place, and that abortion would not follow without proving fatal to the mother. Abortion did not take place, but death supervened in six hours. She was advanced in pregnancy to the fifth month. The death of the fœtus took place in every fatal case but one, before miscarriage. Abortion was the result in only two cases; and one child was born at full time on the third day of the disease. This child is still living, and has in the main good health. On the evening that the child was delivered the mother observed an inflamed spot on her fore-arm, just above the wrist, in which the cellular membrane was involved. When she observed it, she said that she would not live, as all who had been thus affected had died; and her prediction was verified in a few days. The lady who recovered had visited her friends in town, some of whom were affected with the worst forms of the disease. She became but slightly affected, and by taking much care, recovered and gave birth to a fine child, some months afterwards.

Of the contagious nature of this disease I have but little doubt, and what is most singular it propagated the scarlatina anginosa. For instance, a gentleman and lady had that disease, combined with erysipelas, which proved fatal. Their children, who lived at some distance, visited them, and some days after their return home several of them were taken ill with it; but none of them had erysipelas in any form; those, however, of their neighborhoods, who visited them, and became diseased in a similar way, in two or three instances had erysipelas of the face; yet no such disease took place among others in that region, who were not exposed to the contagion.

This same gentleman and lady were visited by a lady who lived in another section of the country; and she also had the affection with erysipelas of the face. Her daughter had the disease combined with erysipelatous inflammation; while several others of the family had it uncombined with that dangerous symptom.

At a number of places through the country, scarlatina existed in its simple or malignant form; but in no place did the same phenomena present themselves as in St. Clairsville and its immediate neighborhood. I saw two cases, however, during the same season, in which two thirds of the nose was involved in an inflammation that had travelled up the schneiderian membrane from the fauces. There was, however, but very little swelling and no vesication. In both cases death was the consequence.

It not unfrequently happens, that a tendency to erysipelatous inflammation is manifested in the eruptive stages of scarlatina, as a result of bleeding or blistering. As an evidence of this I will relate the following case. My friend and former student, the late Dr. W. Wright, of eastern Ohio, informed me, some years ago, that he had, during his residence in Guernsey County, bled a patient laboring under scarlatina, and that the orifice healed in about the common time; but the patient did not recover with the usual rapidity, and in about two weeks the orifice which had seemed entirely well, suddenly ulcerated, and the inflammation rapidly spread and soon involved the whole arm, which terminated in mortification.

We now arrive at the treatment of this disease, which was at least characterised by energy. I usually commenced by bleeding the patient to approaching syncope, after which I subjected him to a warm shower bath; then an antimonial emetic was administered, which was followed by a free cathartic of calomel and jalap, or salts and senna, or some other medicine equally active—always taking care to make calomel one of the ingredients. After a free action of the bowels was effected, a solution of tartar emetic was given every few hours, for the purpose either of keeping the patient nauseated, or with the more useful intention of commanding the circulation, and keeping up an action of the skin. When there was much fever, a repetition of the warm shower was directed every few hours, which was among the very best means used for reducing the morbid heat. When these means did not sufficiently control the febrile action, I had recourse again to the lancet, with mercurial and other purgatives, which were given daily. Indeed, at the commencement of this affection, my inclination and opinion led me to a free use of mercury; but as I advanced in the treatment, I became satisfied that salivation had no beneficial influence, and, indeed, that there did not seem to be any advantage in the exhibition of calomel after the proper action of the abdominal viscera was restored. As there was no ulceration of the fauces, *but a deeply injected condition only*, no gargles were directed but those of a mild character.

To the throat externally, no applications were made, but volatile liniment and emollient poultices. I had long had an abhorrence of blistering in scarlatina, in any form, as I had mostly seen cases where this remedy was used prove fatal, and often, as I thought, from the ill-conditioned state of the blistered surfaces; this was, however, particularly the case in young subjects.

When the disease was mild in its character, my course was very different from the one laid down above: in such cases I did little more than give mild doses of epsom salts, or of oil, with a little calomel, which was given at the onset; and afterwards a light diet was directed with laxatives. I have not unfrequently seen what were obviously mild cases of scarlatina become serious, and end fatally, in consequence of the treatment being so active as to destroy the equilibrium of the circulation, and cause a recession from the surface, with consequent congestions of the brain, abdominal viscera, or other organs, which eventually proved fatal. This result I have occasionally seen after bleeding, when there was much congestion of the skin and subjacent cellular membrane.

Cupping on the temples or back of the neck, had a happy effect in some cases, where there was determination to the brain. I recollect one case in which deep coma existed, that was greatly relieved by cupping. Leeching would no doubt have had a very happy effect; but my location in 1833 prevented any trials being made with it. I did not lose a single case of the simple form of the disease; but the reader knows from the foregoing pages that when a complication with erysipelas took place this fortunate result did not always ensue.

In the treatment of the cases in which erysipelatous inflammation of the face and scalp occurred, but one mode of treatment seemed at all successful; and even that too often failed. The first case of this kind that I met with, was that of a young lady eighteen years of age. She was of a delicate form, and had a feeble constitution. Her disease had progressed some days before I saw her; and about twenty-four hours anterior, an inflamed belt had crossed the nose, and rested on each cheek. This caused alarm in the minds of her friends, that led to a consultation. I found her nearly pulseless, with delirium and subsultus. Cold applications had been used to the inflamed part; and as yet no vesicatiou had taken place, which had probably been prevented by the application of cold water.

The general treatment of this case had been mercurial. Large and repeated doses of calomel had been given, and but little else. Salivation, however, had not supervened. I directed but little to be done, as I saw that death must take place in a few hours.

I was very soon called to another case, which had been managed by the same medical gentleman, and in which the treatment had been similar to that pursued in the above. Calomel had been given largely, notwithstanding the whole face and scalp were involved in the inflammation, which had produced vesication. A tendency to coma and delirium was present. I directed a continuance of the calomel, and applied the mercurial ointment to the vesicated surface. I also wished to bleed the patient, but she would not submit. I gave nauseating doses of antimony, and continued them as long as they could be useful. She however succumbed in a few days.

These cases produced a degree of alarm in my mind, as I felt that in them was a masked form of scarlatina, associated with erysipelas that was calculated to set at defiance the best directed efforts. On mature deliberation, however, I determined on the following course: in the first place, to bleed, in all cases where the patient would bear it, and to repeat it as often as was admissible; in the second place, to purge with calomel and jalap, or senna and salts; and in the third place, to vomit with tartar emetic, either immediately before purging, or within a few hours after. The emetics and cathartics to be repeated according to circumstances; and throughout, antimonials to be given in less than nauseating doses for the purpose of controlling the circulation; whilst blue ointment and poultice should be applied to the blistered surfaces.

This plan I pursued as long as the powers of the system would permit. At this distance of time, I can find but little to regret in my course, unless it be as regards the use of mercurials; for although I occasionally salivated my patients, I am not of the opinion that I saved a single case by it; and if time and reflection can render the judgment more correct, I can now aver that a treatment independent of mercurials would be equally as safe as the contrary. I believe that bleeding, emetics and purgatives, with the constant and untiring use of antimonials from beginning to end, with proper dressings to the vesicated surfaces, were all that were materially useful. To combine calomel with purgatives was possibly useful, but the specific action of mercury seemed to do no good. So far as regards the influence of antimonials in this affection, in preventing a fatal termination, the following case, I presume, will be instructive.

A young lady was taken with scarlatina anginosa, and when I first saw her the inflammation had travelled up the septum narium, and contrary to its usual course appeared at the upper border of the lip, but was rapidly reaching the ridge of the nose. She was bled, and vomited by tartar, which immediately stopped the progress of the inflammation; and it was kept stationary, and eventually removed by the frequent exhibition of nauseating doses of tartar.

Purging will always be resorted to, in those diseases affecting the mucous membrane of the fauces, mouth and nose, because experience has taught most practitioners, that a depletory course (pursued with care) of the stomach and bowels by cathartics has a most happy effect in subduing inflammatory conditions of those parts; hence it is that purgatives of the hydragogue kind are best; such as jalap, epsom salts, &c. When diarrhœa accompanies any form of scarlatina, danger may be apprehended, and Dover's powder, with calomel or blue mass, will be proper. If the head be not affected, other means must be resorted to. In the disease under consideration there was scarcely a case of diarrhœa that came under my observation.

In order to give a just idea of the beneficial effects of bleeding, I will give a short history of two cases; as I think, both were happily terminated in consequence of repeated bleedings, I shall omit the recital of other cases that would equally prove the efficacy of the free use of the lancet.

A gentleman aged 45 was taken with the usual symptoms of the disease, but had no medical attendance for some days, and by various imprudences, erysipelas of the face was established in the usual way. The inflammation involved the whole face and scalp; articulation and swallowing were almost impossible for days; blindness also was produced by the swelling; abscesses formed in the upper eye-lids, and a number on various points of the scalp, each of which when opened contained a tea-spoonful of pus. Vesication was very general and the discharge profuse. Accompanying this condition was pain in the head, general fever, and almost constant delirium.

This patient was bled from the arm three times, about 30 ounces of blood being taken each time, which had a most happy effect; each successive venesection moderating the reaction, and abating the head-ache and delirium. To this remedy was added an antimonial emetic, with continued nauseating doses, and cathartics of calomel and jalap, with dressings to the inflamed surfaces of mercurial ointment and poultices. This patient became convalescent in about ten days from the commencement of the erysipelatous inflammation, and finally recovered. I will cite one case more.

Very near the conclusion of the epidemic, a youth, aged sixteen, complained a little in the evenlng, but went to bed in the usual way. He arose in the night, stepped over the *head* of his bed, and fell about 10 feet. The side of his occiput struck the edge of the lowest step of the stairs, lacerating the soft parts to the bone, and producing a profuse hæmorrhage from the occipital artery.

The patient was instantly thrown into a comatose condition from which he did not recover for five days. During this time I scarcely suspected the presence of any disease other than that produced by the fall; and accordingly treated my patient altogether in a surgical point of light. He was bled twice a day for the first five days; and cathartics and antimonials were administered. The wound was treated in the usual way and cold applications were mostly kept to the scalp.

About the time the comatose condition began to subside, I discovered that erysipelas of the nose was setting in, and I now for the first time found the reason why I had had so much difficulty in controlling the violence of what I had regarded as reaction. This young man had not to suffer the same amount of erysipelatous inflammation that others had, and very soon recovered, having lost about six pounds of blood during the first five days after his fall.

With regard to the treatment pursued in those cases where erysipelatous inflammation of the extremities supervened, but little need be said, as a method was adopted very similar to that pursued in the management of the affections of the head. As a number of cases lingered long, it was a matter of necessity to treat them mildly; and in these cases I tried salivation, but no benefit was derived from it. When suppuration took place the abscesses were opened with the knife, and the wound was dressed in the usual way. Poultices were generally applied to the inflamed parts, and sometimes spirits and water with mercurial ointment.

The pregnant patients were treated actively, particularly as regarded the use of the lancet; for I found that free bleeding relieved pain and of course soothed the last moments of those who sunk beneath the weight of this terrible malady. The single case that recovered was managed without any active treatment. Rest was enjoined, a low diet, and mild purgatives in small doses, with minute portions of tartrate of antimony.

During the prevalence of this disease, I lost in St. Clairsville eight patients by it, and in the country six. The whole number treated by me individually was two hundred, of which one hundred were in town. I saw, however, a considerable number of cases in consultation. I believe that no more than fourteen died in town during the epidemic; all of whom, except one, were adults.

Cincinnati, Oct. 1842.

